# An Incidental Finding of Low-Grade Appendiceal Mucinous Neoplasm in a Case of Borderline Brenner Tumor of the Ovary

**DOI:** 10.7759/cureus.61151

**Published:** 2024-05-27

**Authors:** Priya Dharshini R, Neha Agarwal, Vimal Chander R

**Affiliations:** 1 Pathology, Saveetha Medical College and Hospital, Saveetha Institute of Medical and Technical Sciences, Saveetha University, Chennai, IND

**Keywords:** mucinous neoplasm, low-grade appendiceal mucinous neoplasm, brenner tumor of ovary, borderline brenner tumor, ovarian tumor

## Abstract

The concurrent presentation of a low-grade appendiceal mucinous neoplasm (LAMN) and a borderline Brenner tumor (BT) of the ovary are exceedingly rare. Brenner tumors stand out as a particularly uncommon form, making up only around 5% of all benign epithelial tumors of the ovary. Among the ovarian Brenner, the borderline subtype is even rarer. Appendiceal neoplasm (LAMN) and right ovarian BT cannot be distinguished due to their anatomical position. LAMN is often an incidental finding and at later stages when left undiagnosed may lead to pseudomyxoma peritonei (PMP). This case describes a postmenopausal woman in her 50s experiencing abdominal pain and bloating for a week. Elevated carcinoembryonic antigen (CEA) levels and imaging suggested a potential right ovarian tumor. Interestingly, it revealed a unique combination of borderline Brenner tumor of the right ovary and low-grade appendiceal mucinous neoplasm.

## Introduction

Brenner tumors (BT) are a relatively uncommon subtype of ovarian neoplasm, constituting approximately 1.4 to 2.5% of all such tumors [1]. It is a neoplasm of epithelial origin and is subclassified into three subtypes by the WHO. Most Brenner tumors of the ovary fall within the benign category constituting 95% of the cases [2]. The benign Brenner generally presents as a small, solid, and well-circumscribed nodule measuring less than 2 cm, however, few cases involving larger sizes have also been reported. The asymptomatic presentation of benign Brenner often leads to their incidental detection [1,3]. Therefore, the definitive diagnosis relies upon histopathological examination as the imaging studies typically yield inconclusive results [3]. It presents histologically as nests of transitional cells in a fibrous stroma. The borderline subtype represents less than 5% of all cases of Brenner tumors and primarily occurs in elderly women (typically exceeding 50 years) [2]. These tumors are often unilateral and are typically known for their larger size and often manifest clinically as a palpable abdominal mass that can cause abdominal distension. Histologically, the borderline BT exhibit a characteristic papillary arrangement of uniform stratified neoplastic cells with oval nuclei possessing longitudinal grooves. They further demonstrate a fibro-vascular core and the absence of stromal invasion [4-6]. This histological presentation resembles low-grade papillary urothelial neoplasms [1,7] and frequently includes areas with a benign Brenner component [1,6]. It exhibits both solid and cystic regions, with the solid areas corresponding to the presence of the benign component. They demonstrate a benign clinical course, with local recurrence typically being uncommon. Malignant subtype constitutes less than 1% of all cases of ovarian Brenner tumors [2]. The precise molecular pathway leading to the development of malignant Brenner tumors remains unclear. However, the prevailing theory suggests that these aggressive tumors likely arise from benign or borderline Brenner tumors if not treated initially.

Appendiceal mucinous neoplasms are rare and are incidentally identified in approximately 0.2 to 0.3% of appendicectomy specimens [8]. The WHO classifies these tumors into low-grade appendiceal mucinous neoplasms (LAMN) and high-grade appendiceal mucinous neoplasms (HAMN). Histologically, LAMN is characterized by a villous or flat proliferative mucinous epithelium of intestinal type with low-grade cytologic features. In contrast, HAMN has similar features to LAMN but exhibits high-grade cytological features in addition to micropapillary and cribriform patterns [8,9]. Though the etiology remains unknown, LAMN has a good prognosis. Our comprehensive literature review revealed no documented cases of simultaneous presentation of both borderline Brenner tumor of the ovary and low-grade appendiceal mucinous neoplasm in an individual. The development of these tumors remains independent, and here we report a case of their incidental coexistence.

## Case presentation

**Figure 1:**
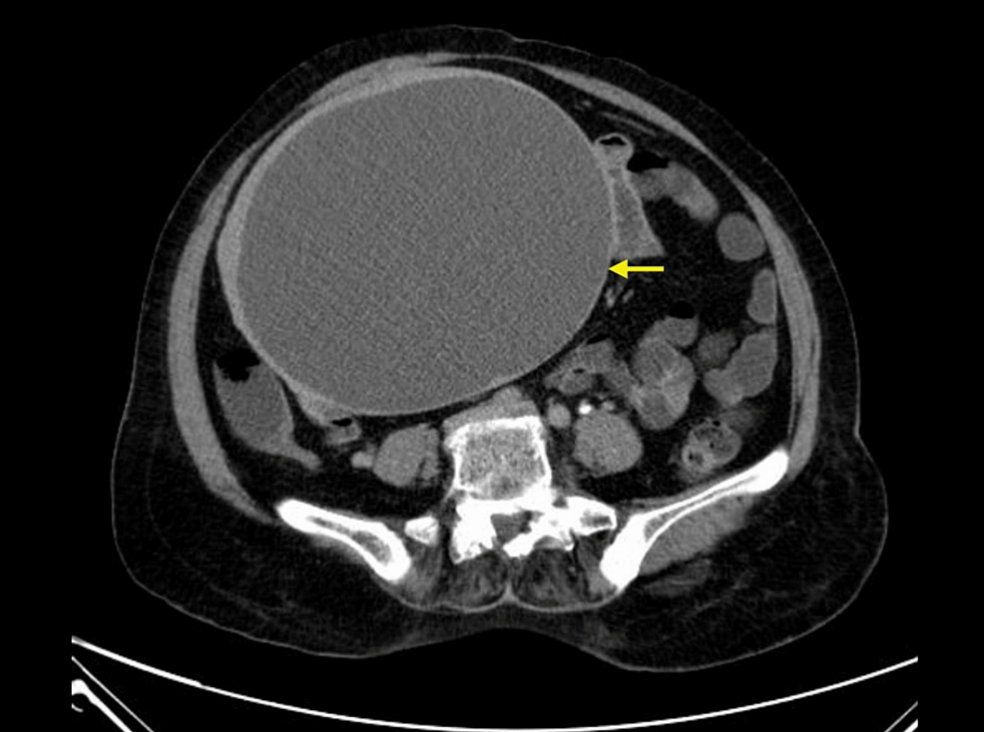
Contrast-enhanced computed tomography (CECT) of the abdomen showing a well-defined cystic lesion involving the right adnexa.

A 55-year-old woman in her post-menopausal state, presented with complaints of abdominal pain and distension for one week. On examination, a freely mobile, bosselated mass of size 25 x 20 cm was palpable occupying the right side of the abdomen. Carcinoembryonic antigen (CEA) was elevated (5.77 ng/ml) and cancer antigen 125 (CA-125) was within normal limits (8.1U/ml). The contrast-enhanced computed tomography (CECT) of the abdomen showed a large, well-defined pelvic-abdominal thin-walled cystic lesion arising from the right adnexa (Figure 1), with peripheral homogenous enhancing solid component possible of ovarian parenchyma. No internal septations were noted in the cystic lesion. These features were indicative of a neoplastic etiology of the right ovary hence the patient was taken for laparotomy. The right ovarian cyst sent for a frozen section showed features of a borderline Brenner tumor. We subsequently received total abdominal hysterectomy with unilateral salpingo-oophorectomy, appendicectomy, peritoneal biopsy, and omentectomy specimens for histopathological examination, and peritoneal wash fluid for cytology.

Gross findings

**Figure 2:**
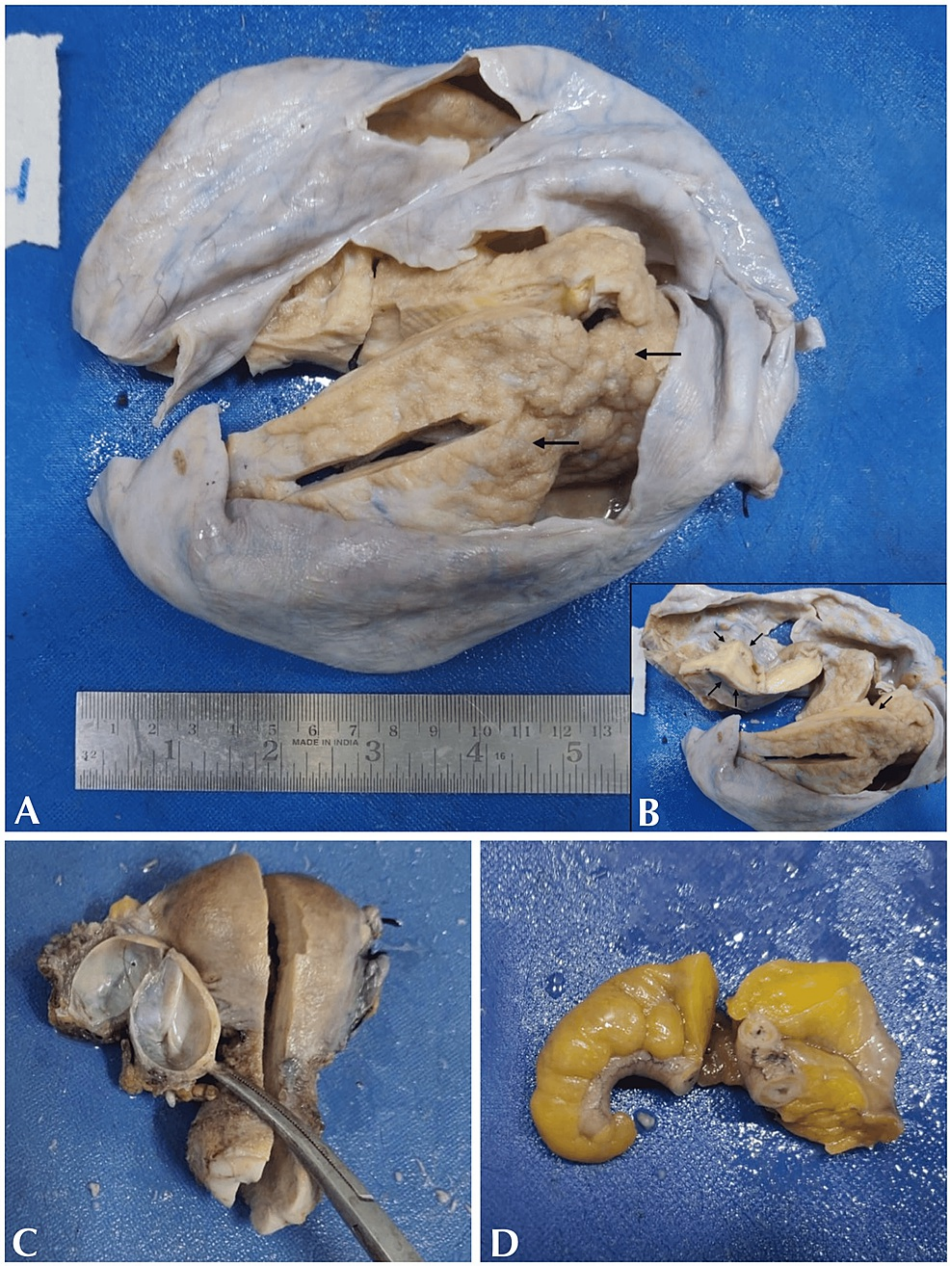
Gross appearance. A. Cut surface of the right ovary showing solid and cystic areas with focal papillary excrescence (arrows) B. Cut section of solid areas. C. Left ovary showing a unilocular cyst. D. Appendix.

Macroscopic examination of the uterus with cervix and bilateral tubes appeared unremarkable. The left ovary measured 3 x 2 x 1.5 cm and the cut surface showed an unilocular cyst of 1.5 cm diameter. The cyst had a smooth inner surface filled with clear fluid (Figure 2C). The right ovary measured 22 x 17 x 13 cm and the cut surface showed solid and cystic areas. The solid areas (Figure 2B) appeared grey-white, and focal papillary excrescences (Figure 2A) were also noted. The appendix with peri-appendicular fat measured 6 cm in length. The external surface was unremarkable and the lumen appeared to be narrowed on the cut surface (Figure 2D). The omentectomy specimen appeared unremarkable.

Microscopy

**Figure 3:**
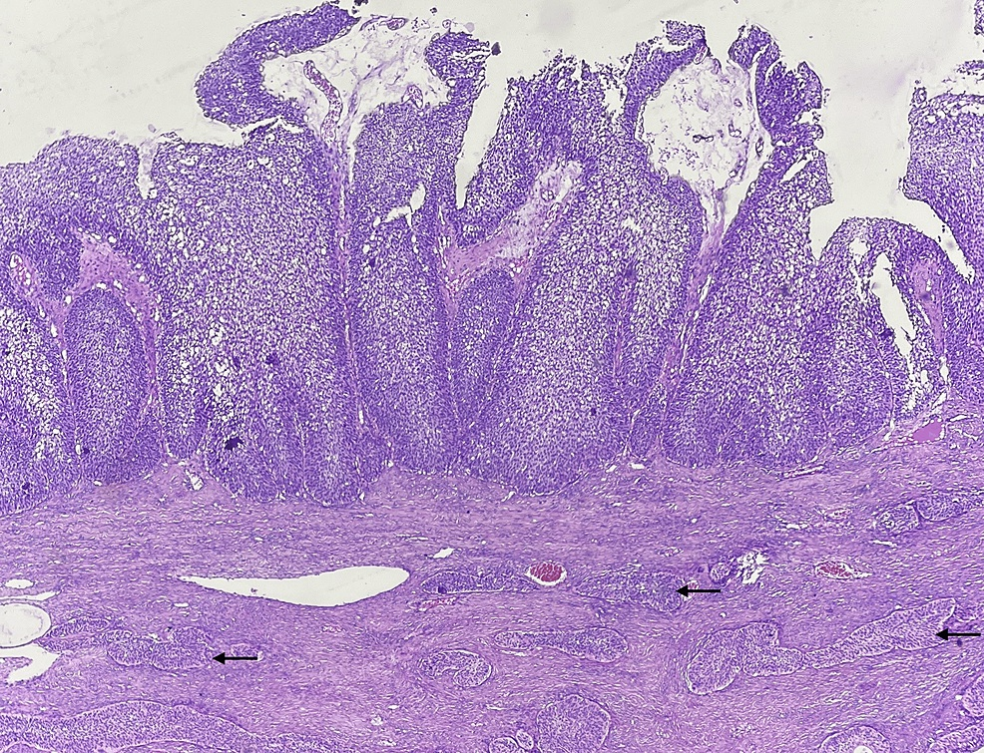
Microscopy of the right ovary on low power magnification showing features of borderline Brenner tumor (BT). Papillary arrangement and solid nests (arrows) of transitional cells in a dense fibrous stroma (H & E).

**Figure 4:**
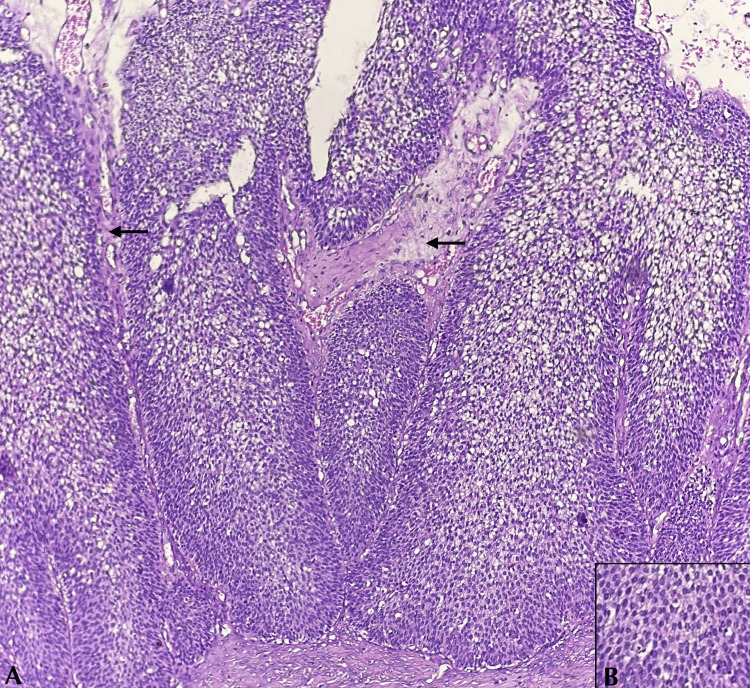
Microscopy of the right ovary on higher magnification. A. Transitional cells in papillary arrangement with fibrovascular core (arrows) B. Individual cells having oval to round nuclei with scant eosinophilic cytoplasm (H & E).

On microscopy, the right ovary showed solid nests and papillary arrangement of transitional cells (Figure 3) having oval nuclei with scanty eosinophilic cytoplasm (Figure 4B) surrounded by a dense fibrous stroma with no evidence of stromal invasion.

**Figure 5:**
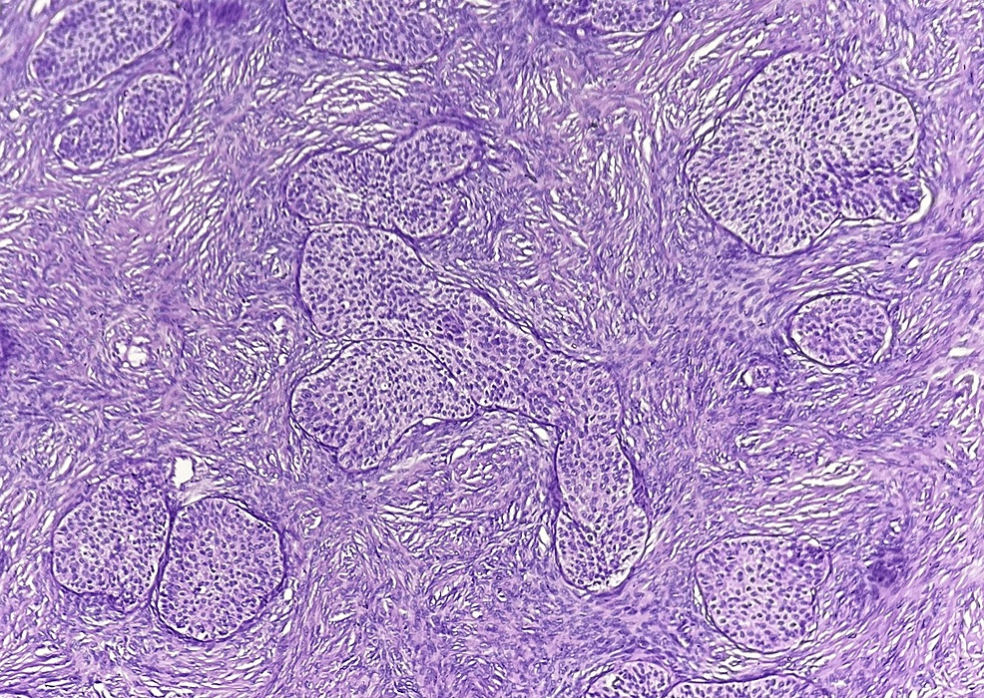
Microscopy of the left ovary on higher magnification showing benign Brenner features. Transitional cell nests in a dense fibrous stroma (H & E).

**Figure 6:**
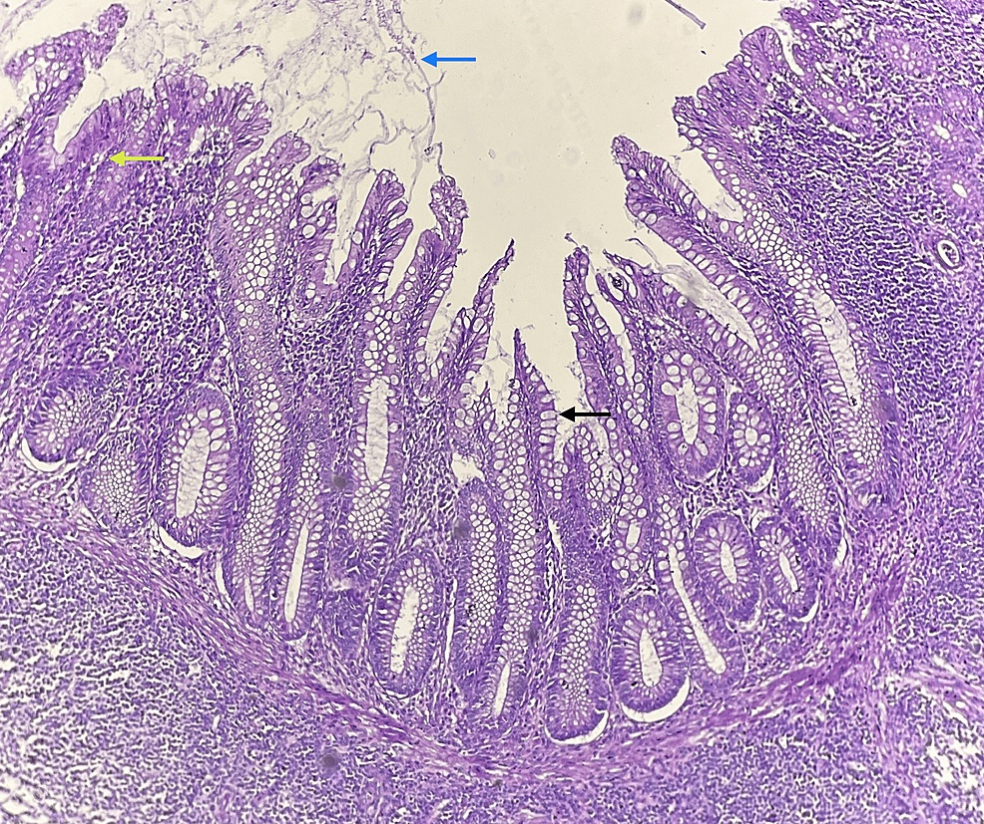
Microscopy of the appendix on higher magnification showing features of low-grade appendiceal mucinous neoplasm (LAMN). Appendiceal lumen showing mucinous material (blue arrow), lined by mucinous columnar epithelium with apical mucin (black arrow), and a focal area showing nuclear pseudostratification (yellow arrow) (H & E).

The left ovary showed transitional cells in solid nests surrounded by a dense fibrous stroma with no stromal invasion (Figure 5). The uterus, cervix, and bilateral tubes appeared unremarkable. Microscopy of the appendix showed an appendiceal wall with a dilated lumen filled with mucinous material. The mucosa is lined by a layer of columnar epithelium with apical mucin and round to oval vesicular nuclei with focal areas showing pseudostratification of the nuclei (Figure 6). The peritoneal biopsy and the omentum were free of tumor. The fluid from the peritoneal wash was negative for malignant cells. 

A final diagnosis of borderline Brenner tumor of the right ovary, benign Brenner tumor of the left ovary, and low-grade appendiceal mucinous neoplasm of the appendix were made. The patient recovered uneventfully and was subsequently discharged and is on regular follow-up.

## Discussion

Ovarian neoplasms of epithelial origin represent the predominant subtype encountered among ovarian tumors. In contrast, the Brenner tumors are a comparatively infrequent subset of ovarian epithelial tumors [10]. The prevailing hypothesis suggests an origin of Brenner tumors in Walthard cell nests, as they share similar morphological and immunohistochemical profiles, positive for GATA3 expression, and absence of PAX2, PAX8, SALL4, and WT1 expression [1,6,11]. These Walthard cell nests are innocuous aggregates of transitional epithelium, often encountered in the fallopian tube or some cases in the ovarian hilum [1,11]. However, studies have suggested further investigation since the aforementioned markers do not constitute a unique identifier for Walthard nests [1,6]. The incidence of borderline and malignant Brenner tumors increases with age compared to the benign subtype [3] as it is believed that borderline tumor tends to arise from the benign counterpart [1]. Pathogenesis of the Brenner tumor is unclear but the loss of p16 is thought to play a major role in the progression of the tumor from benign to the borderline and malignant variant [6]. p16 serves as a cell cycle inhibitor protein, preventing abnormal cell growth and proliferation by binding to complexes of cyclin-dependent kinases [12]. The decrease or absence of p16 protein expression observed in both borderline and malignant Brenner tumors with retained p16 expression in benign subtypes suggests that the loss of p16 may contribute to the progression from benign to borderline and malignant subtypes [6]. Furthermore, studies have demonstrated that the activation of the EGFR pathway and downstream co-activation of the Ras-MAPK pathway may also drive the progression [13]. 

The benign Brenner tumor of the ovary typically presents without associated symptoms and is frequently diagnosed incidentally as a small, solid ovarian mass. In contrast to their benign counterpart, borderline and malignant Brenner tumors of the ovary exhibit a distinct presentation. These tumors typically manifest as larger masses with a heterogeneous composition, characterized by the presence of both solid and cystic areas. The Brenner tumor of the ovary mostly involves a single ovary whereas the malignant subtype is more commonly known to have a bilateral involvement [1]. However, in our case, we had bilateral ovarian involvement with borderline features of one ovary and benign features of Brenner in the other ovary. Borderline Brenner tumor carry a significantly more favorable prognosis compared to malignant Brenner tumor of the ovary.

Primary appendiceal neoplasms are uncommon, constituting less than 1% of all gastrointestinal tumors, and are frequently discovered incidentally [14]. Furthermore, LAMN is identified in only 0.7 to 1.7% of all cases of appendicectomies indicating their rarity [15]. While the etiology of LAMN is unclear, these tumors frequently harbor KRAS and GNAS mutations [9]. In contrast, HAMN less often shows GNAS mutations, suggesting these tumors may have distinct origins as evidenced in the 5th edition of the WHO. Appendiceal neoplasms are identified incidentally following appendicitis or other abdomen-related clinical presentations leading to surgery. Approximately 15 to 20% of LAMN diagnoses arise incidentally, the majority of which are identified during surgical procedures undertaken for the above-mentioned unrelated conditions [16]. In our case, the patient had a short history of abdominal distension and pain for one week with radiological evidence of a tumor involving the right ovary, the contemporaneous appendiceal neoplasm was obscured. Like in our case, most of the appendiceal neoplasm goes unnoticed in an underlying ovarian pathology due to its anatomical positioning [17]. Undiagnosed LAMN in the later stages may rupture and lead to pseudomyxoma peritonei (PMP), which is a rare complication with a high mortality rate. PMP usually occurs following the rupture of the mucinous tumors primarily involving the appendix or the ovary. The mucinous material from the tumors following the rupture leads to intraperitoneal dissemination resulting in PMP [15-18].

Low-grade appendiceal mucinous neoplasms are more frequently seen to be associated with an underlying mucinous neoplasm of the ovary [17]. Brenner tumor of the ovary generally shows a strong association with mucinous elements involving the same ovary. These mucinous elements may vary from the presence of mucinous glands to mucinous cystic tumors [6,10,11]. However, in our patient, the mucinous neoplasm was observed in the appendix, rather than in the ovary. Early diagnosis and intervention are crucial for maximizing favorable outcomes in both low-grade appendiceal mucinous neoplasm and borderline Brenner tumor of the ovary. In the case of LAMN, this proactive approach helps prevent potential complications like pseudomyxoma peritonei. Similarly, for an ovarian borderline Brenner tumor swift action minimizes the risk of progression to a more aggressive malignant form. Thus, surgical intervention is the primary treatment modality for both borderline Brenner tumors and LAMN. This approach aims to achieve complete resection of the lesion. In the management of LAMN, the focus lies on preventing complications. Post-operative surveillance for LAMN patients typically involves radiographic imaging at six-month intervals for the initial two years following appendicectomy. This monitoring strategy aims to detect any potential tumor recurrence or PMP-related complications. The five-year survival rate for patients with localized LAMN remains high at approximately 95% [19]. Therefore, identification of LAMN and BT in their early stages is essential for improved patient outcomes.

## Conclusions

Borderline Brenner tumor of the ovary and low grade appendiceal mucinous neoplasm are rare types of neoplasms even when present individually, making this combination a very uncommon presentation. In women with symptoms of abdominal pain (right side) even with radiological evidence of an ovarian pathology, appendicular pathologies may also be considered. Such appendicular mucinous neoplasm may not be restricted to borderline mucinous neoplasms alone but can coexist with other subtypes of ovarian neoplasm.

## References

[1] Alloush F, Bahmad HF, Lutz B, Poppiti R, Recine M, Alghamdi S, Goldenberg LE (2023). Brenner tumor of the ovary: a 10-year single institution experience and comprehensive review of the literature. Med Sci (Basel).

[2] Jia Y, Zhang S, Ge Y, Bai F, Zhu Z, Li F, Jia S (2024). Imaging features and differential diagnosis of benign and borderline/malignant ovarian Brenner tumor. J Radiat Res Appl Sci.

[3] Alamer LA, Almukhadhib OY, Al Zahrani KA, Adham M, AlMousa RA (2023). Ovarian Brenner tumor: a report of two cases and literature review. Cureus.

[4] Salibay CJ, Zanfagnin V, Miller H, Walia S, Brunette LL, Wang T (2021). Borderline Brenner tumor of the ovary coexisting with an ovarian mucinous cystadenoma with focal atypical epithelial proliferation: a rare case with review of the literature. Int J Surg Pathol.

[5] De Cecio R, Cantile M, Collina F (2014). Borderline Brenner tumor of the ovary: a case report with immunohistochemical and molecular study. J Ovarian Res.

[6] Zheng R, Heller DS (2019). Borderline Brenner tumor: a review of the literature. Arch Pathol Lab Med.

[7] Costeira FS, Félix A, Cunha TM (2022). Brenner tumors. Br J Radiol.

[8] Li X, Zhou J, Dong M, Yang L (2018). Management and prognosis of low-grade appendiceal mucinous neoplasms: a clinicopathologic analysis of 50 cases. Eur J Surg Oncol.

[9] Yanai Y, Saito T, Hayashi T (2021). Molecular and clinicopathological features of appendiceal mucinous neoplasms. Virchows Arch.

[10] Yüksel D, Kılıç C, Çakır C (2022). Brenner tumors of the ovary: clinical features and outcomes in a single-center cohort. J Turk Ger Gynecol Assoc.

[11] Roma AA, Masand RP (2014). Ovarian Brenner tumors and Walthard nests: a histologic and immunohistochemical study. Hum Pathol.

[12] Foulkes WD, Flanders TY, Pollock PM, Hayward NK (1997). The CDKN2A (p16) gene and human cancer. Mol Med.

[13] Zanfagnin V, Lee T, Zhao C, Wang T (2023). Advances in diagnosis, clinical management and molecular characterization of ovarian Brenner tumors. Gynecol Obstet Clin Med.

[14] Bahmad HF, Aljamal AA, Alvarez Moreno JC, Salami A, Bao P, Alghamdi S, Poppiti RJ (2021). Rising incidence of appendiceal neoplasms over time: does pathological handling of appendectomy specimens play a role?. Ann Diagn Pathol.

[15] Perivoliotis K, Christodoulidis G, Samara AA (2021). Low-grade appendiceal mucinous neoplasm (LAMN) primarily diagnosed as an ovarian mucinous tumor. Case Rep Surg.

[16] Misdraji J (2015). Mucinous epithelial neoplasms of the appendix and pseudomyxoma peritonei. Mod Pathol.

[17] Muramoto T, Koike R (2021). Synchronous mucinous borderline tumor of the ovary and low-grade appendiceal mucinous neoplasm. Open J Obstet Gynecol.

[18] Wang AS, Ismael HN, Parikh J, Modesto VL (2022). Low-grade appendiceal mucinous neoplasm: a case series. Cureus.

[19] Al-Tarakji M, Ali SM, Shah AA, Petkar MA, Mirza S, Singh R, Zarour A (2020). A unique case of low-grade mucinous neoplasm in stump appendectomy. Case Rep Surg.

